# Evidence of disrupted high-risk human papillomavirus DNA in morphologically normal cervices of older women

**DOI:** 10.1038/srep20847

**Published:** 2016-02-15

**Authors:** Sarah M. Leonard, Merlin Pereira, Sally Roberts, Kate Cuschieri, Gerard Nuovo, Ramanand Athavale, Lawrence Young, Raji Ganesan, Ciarán B. Woodman

**Affiliations:** 1 School of Cancer Sciences, College of Medical and Dental Sciences, University of Birmingham, Edgbaston, Birmingham, B15 2TT, UK; 2 Scottish HPV Reference Laboratory, Royal Infirmary of Edinburgh, Edinburgh EH3 6DT, UK; 3 The Ohio State University, Comprehensive Cancer Center, OH 43210, United States; 4 University Hospitals Coventry and Warwickshire, Clifford Bridge Road, Coventry, CV2 2DX, UK; 5 Warwick Medical School, University of Warwick, CV4 8UW, UK; 6 Birmingham Women’s NHS Foundation Trust, Mindelsohn Way, Edgbaston, Birmingham, B15 2TG, UK

## Abstract

High-risk human papillomavirus (HR-HPV) causes nearly 100% of cervical carcinoma. However, it remains unclear whether HPV can establish a latent infection, one which may be responsible for the second peak in incidence of cervical carcinoma seen in older women. Therefore, using Ventana *in situ* hybridisation (ISH), quantitative PCR assays and biomarkers of productive and transforming viral infection, we set out to provide the first robust estimate of the prevalence and characteristics of HPV genomes in FFPE tissue from the cervices of 99 women undergoing hysterectomy for reasons unrelated to epithelial abnormality. Our ISH assay detected HR-HPV in 42% of our study population. The majority of ISH positive samples also tested HPV16 positive using sensitive PCR based assays and were more likely to have a history of preceding cytological abnormality. Analysis of subsets of this population revealed HR-HPV to be transcriptionally inactive as there was no evidence of a productive or transforming infection. Critically, the *E2* gene was always disrupted in those HPV16 positive cases which were assessed. These findings point to a reservoir of transcriptionally silent, disrupted HPV16 DNA in morphologically normal cervices, re-expression of which could explain the increase in incidence of cervical cancer observed in later life.

Although the United Kingdom NHS Cervical Screening Programme (NHS CSP) has for many years achieved excellent coverage in women aged between 50 and 64 (~80%), one in five cancers still occur in women aged 65 or older; and 49% of all deaths from cervical cancer are in this age-group^1^,^2^.

At one time it could be said that the vast majority of these cases occurred in unscreened women. However, a recent NHS CSP audit which included 1341 cervical cancers diagnosed in women aged 65–83 suggested that this is no longer the case. Many of these women exited the screening programme with negative smear tests; 44% had at least three negative smears after the age of 50^1^. These screening histories are consistent with a recent study from Kaiser Permanente, Northern California which included 56 women with cervical cancer aged 65 or older; 25% of these women were found to have had three consecutive negative smear tests between the ages of 55 and 65^3^.

How do we explain the development of cervical cancer in women who exit the screening programme at 65 with a history of normal smear tests over the preceding fifteen years? It has been argued that incident cervical HPV infections are uncommon in women over the age of 65 and that in any event, the extended natural history of cervical neoplasia makes it improbable that these infections would have sufficient time to progress to invasive cancer. Therefore, we can only conclude that many of these women already had at the time of leaving the screening programme, a cervical HPV infection which was uncoupled from those cytopathic changes associated with virus infection in younger women. One possible explanation for this timeline would be the reactivation of HPV infection from a quiescent or latent state during which productive viral infection is suspended.

Although recent epidemiological studies offer circumstantial evidence supporting a natural history of HPV infection which includes such a latent state, the virological characteristics of a latent cervical HPV infection have yet to be defined^4^,^5^. The most compelling evidence for HPV latency comes from *in vivo* studies using the rabbit oral papillomavirus model. These experiments point to a model of virus latency in which papillomaviruses retained under immunological control in the basal epithelial stem cell pool can be induced to reactivate periodically^6^. Given that we now have a highly HPV exposed and ageing population there is a need for a clearer understanding of the potential for virus reactivation in later life and the implications this might have for our current cancer prevention and managment strategies.

Towards this end, we have measured, using sensitive *in situ* hybridisation methods, quantitative PCR assays and viral and cellular biomarkers of productive and transforming viral infection, the prevalence and characteristics of HPV genomes in cervical material taken from a cohort of women undergoing hysterectomy for reasons unrelated to epithelial abnormality. Based on the observations made in animal models we expected to find a low copy number of viral episomes in the basal epithelial cell compartment with evidence of early viral gene expression in the absence of a productive infection. What we found was quite different.

## Materials and Methods

### Study population

**Figure 1:**
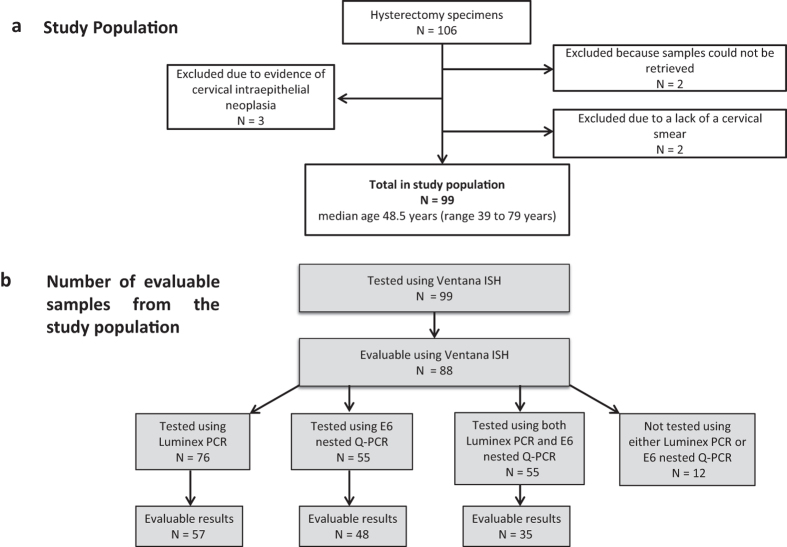
Summary of cohort. (**a**) Flow diagram showing the number of samples which were initially excluded from our study population. (**b**) Number of samples which were tested initially for HPV using ISH and then further confirmed for the presence of HPV using either Luminex PCR or nested HPV16 E6 PCR. For 12 samples neither PCR assay was performed due to the unavailability of DNA. Luminex PCR was performed on 76 samples and nested HPV16 E6 PCR was performed on 55 of the same samples. Following amplification, evaluable results were available for 57 samples tested with Luminex PCR and 48 for those tested with nested HPV16 E6 PCR. Evaluable results were available for 35 samples which had been tested with both assays.

This comprised 106 consecutive patients (median age 48.5 years, range 35–79 years) who had a hysterectomy performed between 2006 and 2009 at the University Hospital Coventry and Warwickshire NHS Trust for reasons unrelated to uterine malignancy and who were reported to have no histological evidence of epithelial abnormality of the cervix. Hysterectomy samples including both abdominal and vaginal hysterectomies but not sub-total hysterectomies were included in our study. For all samples in our cohort the endocervical canal and cervical transformation zone is represented. Fully informed written consent was obtained from each patient and all experiments were performed in accordance with relevant guidelines and regulations. Approval for this project was given by the National Research Ethics Service, West Midlands - Coventry & Warwickshire (ethics ref no: 04/Q2802/104). Seven patients were excluded from subsequent analyses following further independent histological review undertaken by two of the authors (MP, RG). Cytological histories of women undergoing hysterectomy were obtained from the “Open Exeter National Database System” which holds the records of women attending the NHS Cervical Screening Programme. Cytological histories were recorded as either negative, borderline nuclear changes, mild dyskaryosis or severe dyskariosis^7^. A summary of the study population, samples removed and samples tested is shown in Fig. 1.

### Preparation of study material

Paraffin embedded blocks were sectioned every 4 μm and one section stained with haematoxylin and eosin; sequential sections were used for routine immunohistochemical staining and *in situ* analyses with the last of these sections used for HPV DNA testing. To avoid cross contamination, the microtome blades were changed between the cutting of each block. DNA was extracted using AllPrep DNA/RNA FFPE (formalin fixed paraffin embedded) kit, according to manufacturer’s instructions (Qiagen) and stored at −20 °C.

### *In situ* hybridisation (ISH)

The INFORM HPVIII Family 16 Probe(B) which is capable of detecting 12 oncogenic HPV genotypes (Ventana Medical Systems) was used to stain cervical tissues. Untransfected and HPV18 genome transfected human keratinocytes grown as organotypic rafts were used as negative and positive controls^8^, in addition to the HPV positive and negative xenograft tissues provided by Ventana. A blue reaction product, either in the form of diffuse nuclear and cytoplasmic or punctate nuclear staining, was accepted as positive hybridisation signals. Three pathologists independently reviewed the ISH slides (MP, RG and GN) and agreed on the results for all cases.

Eleven cases were excluded from this analysis; 6 because of high background staining in non-epithelial and endocervical cells and in the cytoplasm of squamous epithelia; and 5 because of significant squamous epithelial disruption; 88 evaluable cases remained. Two other ISH methods were evaluated during this study, GenPoint DAKO HPV DNA ISH assay, Abbott Vysis Cervical FISH HPV DNA ISH assay and Bond^TM^ DNA ISH assay; these were optimised on organotypic raft sections of untransfected and HPV18 genome transfected keratinocytes but discarded because of high background staining and low sensitivity after testing on archival FFPE cervical tissue sections.

### PCR based assays

#### DNA genotyping

HPV genotyping was performed at the Scottish HPV Reference Laboratory, Edinburgh using the Multimetrix assay (Diamex) which utilises Luminex^®^ technology. The assay offers individual typing of 24 HPV types with β-globin as an endogenous control. Cases were classified as invalid when β-globin and HPV were absent. A negative control containing no genomic DNA was included to confirm the absence of contamination.

#### Nested HPV16 E6 Q-PCR

Fifty-five samples were tested for the presence of HPV16 DNA using nested Q-PCR primers for E6. The primer and probe sequences used in this analysis are shown in Supplementary Table S1. For the outer PCR reaction, 8 μl of cervical DNA was amplified in a gradient thermocycler (Eppendorf) using 12.5 μl of 2X PCR master mix (Promega) and 10 pmol of outer PCR primers under the cycle conditions; 95 °C for 15 min, followed by 60 cycles of 95 °C for 30 s, 54 °C for 45 s and 72 °C for 1 min. For the inner Q-PCR reaction, 5 μl of product was amplified in a 7900HT Fast Real-Time PCR System (Applied Biosystems) using 12.5 μl Taqman master mix (Applied Biosystems), 10 pmol of inner primers and 2.5 pmol of probe. Assays were performed in triplicate under the cycle conditions; 50 °C for 2 min, 95 °C for 12 min, followed by 40 cycles of 95 °C for 15 s and 55 °C for 30 s. DNA extracted from primary human keratinocytes (PHK) containing episomal HPV16^9^, as well as HPV16 DNA positive cervical cancer cell lines SiHa and CaSki were used as positive controls. The HPV negative cervical cancer cell line C33A was used as a negative control. The water control amplified in the first PCR reaction was carried through to the second Q-PCR reaction to control for PCR product contamination.

#### Nested PCR for HPV16 E2

The integrity of the *E2* gene was assessed using overlapping primers that spanned the full length of the HPV16 *E2* gene using primers shown in Supplementary Table S1. For the outer PCR reaction, 8 μl of cervical DNA was amplified in a gradient thermocycler (Eppendorf) using 12.5 μl of 2X PCR master mix (Promega) and 10 pmol of outer PCR primers under the cycle conditions; 95 °C for 15 min, followed by 60 cycles of 95 °C for 30 s, 54 °C for 45 s and 72 °C for 1 min. For the inner PCR reaction, 5 μl of product was amplified in a gradient thermocycler (Eppendorf) using 12.5 μl of 2X PCR master mix (Promega) and 10 pmol of inner PCR primers under the cycle conditions; 95 °C for 15 min, followed by 40 cycles of 95 °C for 30 s, 55 °C for 45 s and 72 °C for 1 min. PCR products were electrophoreised on a 2% agarose gel alongside 1 μg of 100 bp ladder (promega). DNA extracted from PHK containing episomal HPV16 and SiHa cells which contains integrated HPV16 were used as controls for intact and disrupted *E2* repectively. The water control amplified in the first PCR reaction was carried through to the second PCR reaction to control for PCR product contamination.

### Immunohistochemistry

#### HPV16 E4 expression

Ten cases which tested positive for HPV16 using Ventana ISH and either HPV16 nested PCR or Luminex were also tested for HPV16 E4 protein expression using the E4 monoclonal antibody 1D11 by immunohistochemistry^10^. Two cases which tested negative for HPV16 were used as negative controls and tissue from a patient with cervical intraepithelial neoplasia type 2 (CIN 1) was used as a positive control^11^.

#### p16 INK4a expression

All cases were tested for p16 INK4a antigen expression using the monoclonal mouse antibody (clone E6H4, CINtec^®^ Histology Kit-9511). Organotypic raft tissue of HPV18 transfected keratinocytes and tissue from a patient with koilocytosis were used as positive controls. Sections of organotypic rafts generated from untransfected PHK were used as a negative control. Block-like homogeneous staining involving full thickness of the cervical epithelium (basal, supra-basal and intermediate layers) was considered evidence of p16 INK4a protein over-expression, whereas focal staining of clusters or isolated cells was disregarded^12^.

### Statistical Methods

Non-parametric Wilcoxon rank-sum tests were carried out to determine whether the observed differences in age between the Ventana positive and negative groups were statistically significant. Cross-sectional comparisons of the prevalence of HR-HPV in study samples were made using contingency tables, with estimates of relative risk obtained by unconditional maximum likelihood estimation and the associated 95% confidence intervals constructed using a normal approximation; tests of hypotheses were undertaken using Pearson’s chi-squared χ2 test with continuity correction. Analyses associating the presence of HPV with a history of cytological abnormality were performed using statistical methods described by Collins *et al.* 2009^13^. Analyses were performed using Stata version 11.1 software (StataCorpLP), and a p-value of <0.05 was considered statistically significant.

## Results

### Detection of HPV16 DNA in normal cervices

#### ISH results

**Figure 2:**
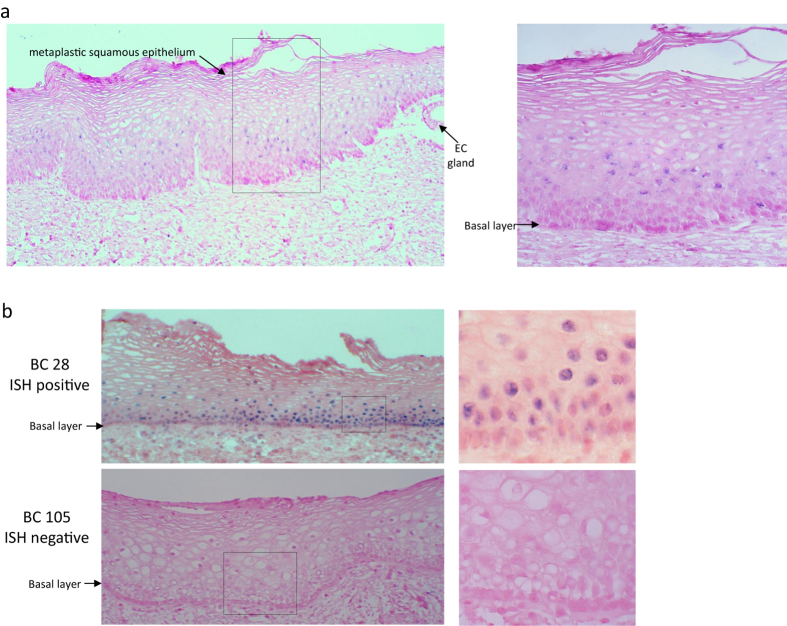
Ventana *in situ* hybridization in benign cervical samples. (**a**) A representative example of Ventana ISH showing the presence of HPV in the transformation zone (BC 54); (EC - endocervical gland). The left panel shows the results from Ventana ISH staining x 10 magnification and the right panel shows a higher magnification (60X) of the region indicated by a box. (**b**) Representative examples of a HPV positive (BC 28) and a HPV negative (BC 105) benign cervix sample. The left panel shows the results from Ventana ISH staining x 20 magnification and the right panel shows a higher magnification (60X) of the region indicated by a box. In sample BC 28, blue punctate staining is observed in the nuclei of basal and suprabasal cells.

Of the 88 evaluable cervices screened using Ventana ISH, 37 (42%) tested positive and 51 (58%) tested negative for HPV DNA, with a punctate nuclear staining pattern observed for each HPV positive case (Fig. 2, Supplemental Fig. 1). In the cervices which tested positive, HPV was detected in the transformation zone (TZ) of 32 cervices (Fig. 2a). The remaining 5 cervices had a positive signal in the outer ectocervix (Fig. 2b). Although HPV was predominantly detected in the TZ, the distribution of HPV DNA varied throughout the epithelium from case to case, and was not always detected in the basal layers. When we compared the median age between HPV negative (49 years – range 36–79 years) and HPV positive women, (52 years – range 35–81 years), we found no significant difference (Wilcoxon rank sum test, p = 0.8591).

#### Validation of ISH results using PCR based methods

Of 76 cervices which were also tested using the Luminex assay, 57 generated a valid result (when β-globin and/or HPV was present): compared to ISH negative samples, ISH positive samples were significantly more likely to test positive for HPV using Luminex (29.6% vs. 0.0% x^2^ = 10.34: 1 df: p < 0.001) (Table 1). Of 55 cervices which were also tested using a nested HPV16 E6 Q-PCR assay, 48 provided a conclusive result: compared to ISH negative samples, ISH positive samples were more than three times as likely to test positive for HPV16 using E6 Q-PCR (75% vs. 21%: RR = 3.3 95% CI 1.6 to 6.7) (Table 1). When we examined the overlap between the assays used to test for HPV, we did not find an exact concordance between the three tests (Table 2). A number of studies have examined the concordance between Ventana ISH and PCR based detection systems and have found that precise concordance was not achieved even though these results were generated from HPV testing in tumours or pre invasive disease specimens which have a high viral load^14^,^15^,^16^. Our Ventana results show that HPV positive cells are scanty and focal in nature in these normal cervices, thus detection of HPV using PCR based methods would be more difficult due to the small number of HPV positive cells in a background of HPV negative cells, therefore complete agreement between the Ventana ISH and PCR assays would not have been anticipated.

#### Association with history of cytological abnormality

A cervical smear history prior to hysterectomy was available for 83 women of whom 22 had a history of cytological abnormality [median time from last abnormal smear to hysterectomy = 11.5 years (range 1–22 years)]. Those women who tested positive using ISH were nearly three times as likely to have a history of any cytological abnormality (41.7% vs. 17.5%: RR = 2.8 95% CI 1.4–7.6). When this analysis was repeated exluding borderline nuclear abnormalities and retaining only definite dyskaryosis, the RR increased to 9.1 (95% CI 1.2 to 70.1).

### No evidence of a productive or a transforming infection in HPV16 DNA positive normal cervices

**Figure 3:**
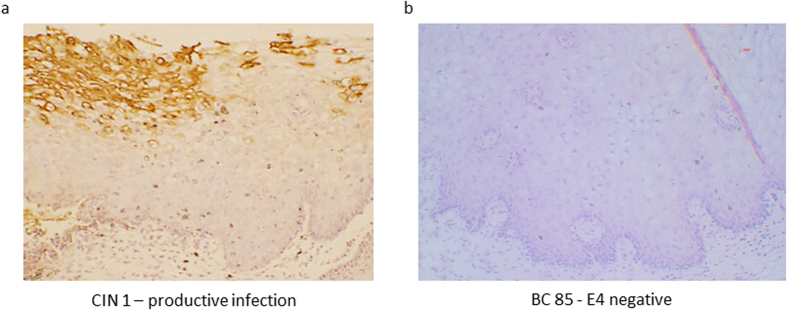
Absence of E4 staining in benign cervical samples. (**a**) E4 staining in a case of CIN 1 served as a positive control. (**b**) A representative example of an E4 negative cervical sample (BC 85) which was positive for HPV using ISH and Q-PCR.

To establish if HPV16 DNA positivity was associated with a productive viral infection, 10 tissue sections which tested positive for HPV16 DNA using Ventana and at least one other HPV detection method, were stained for the viral biomarker E4, that is highly expressed in productive HPV infections and that marks the initiation of the vegetative stage of the virus life cycle (Supplemental Table S2)^17^. While the CIN 2 control tissue stained strongly for E4, there was no evidence of productive infection in any of the normal cervical tissue (Fig. 3).

Deregulation of HPV16 oncoprotein expression is associated with increased levels of the cellular biomarker p16INK4a. No evidence of a block-like homogeneous p16INK4a staining pattern involving the full thickness of the cervical epithelium which is indicative of HPV induced dysplasia^18^ was found in any of the HPV16 positive or negative samples (data not shown).

### Disruption of the *E2* gene in HPV16 DNA positive normal cervices

**Figure 4:**
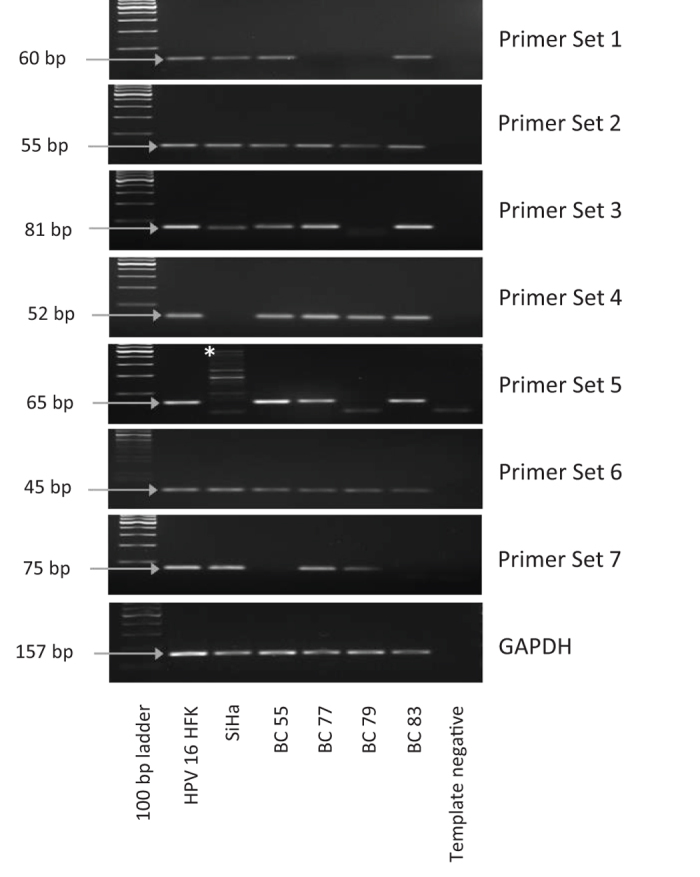
Representative examples of HPV16 E2 disruption in benign cervix samples. HPV16 human foreskin keratinocytes (HFK) and SiHa represent amplification from HPV16 episomal and integrated controls; an intact E2 gene in primary HFK transfected with episomal forms of HPV16; a disrupted E2 gene in the HPV16 positive cervical cell line, SiHa. Results from each of the 7 overlapping primer sets and housekeeping gene (GAPDH) are shown for each of the 4 benign cervix samples (BC 55, BC 77, BC 79 and BC 83); with the E2 gene disrupted in all 4 patient samples. A template negative PCR reaction served as a negative control. * Multiple bands are sometimes observed if the concentration of DNA going into the second PCR reaction is high.

To determine if the HPV16 DNA detected in the normal cervices was present in episomal forms, the integrity of the E2 gene was investigated by nested PCR using a series of overlapping E2 primers. Nested PCR was performed on all 18 samples which tested positive for HPV using Ventana and Q-PCR (Supplemental Table S2). Disruption of the HPV16 *E2* gene was detected in all samples tested; illustrative examples are shown in Fig. 4. The absence of a PCR product at the correct size on the gel excludes the possibility of episomal HPV16 being present.

## Discussion

Our findings point to a reservoir of transcriptionally silent HPV16 in normal cervical epithelium removed from older-women undergoing hysterectomy for reasons unrelated to cervical neoplasia. Our findings offer the first possible scientific explanation for the cause of the second peak of cervical cancer incidence that occurs in older women. As HPV16 could only be detected in these cervices in a disrupted form, the persistence of virus in these samples cannot be attributed to a latent infection as usually defined^6^. Implicit in the definition of viral latency is the capacity to synthesise new viral progeny. HPV genome integration marks the end of the virus life cycle and the loss of this capacity, consistent with the absence of HPV16 E4 expression, a viral biomarker of productive infection, in these samples. While we cannot exclude the possibility that low levels of viral oncogenes are expressed from the disrupted forms of HPV16 DNA, the absence of diffuse immunostaining for p16INK4a and the absence of any cytopathic effect, suggests that these levels are insufficient to establish a transforming infection. Therefore, functionally, at least, the viral DNA could be considered transcriptionally silent. Whilst disruption of the *E2* gene is a strong indicator of viral DNA integration, the assay we used does not identify the viral-cellular junction^13^,^19^; our future studies will confirm the physical status of the viral DNA in normal cervices. Although silent HPV integrants have been found alone or alongside transcriptionally active integrants in cervical cancers and in CIN^20^,^21^,^22^,^23^, we believe we have provided the first robust estimate of the prevalence of silent HPV16 DNA in morphologically normal cervices of older women.

Our findings help illuminate a number of previously puzzling aspects of the exposure-disease relationship in older women^24^,^25^,^26^. The observation that HPV positive older women are less likely to have a contemporaneous abnormal smear than HPV positive younger women could reflect the emergence in later life of integrated but transcriptionally silent high-risk HPV types. Re-expression in later life of viral oncogenes from transcriptionally silent HPV integrants could explain why the risk of cervical cancer continues to increase throughout a woman’s life-time after adjustment for hysterectomy rates^27^,^28^ and to accelerate after age 60 years in those who appear to have been adequately treated for CIN earlier in life. It could also explain why some women go on to develop cervical cancer having exited the screening programme at the age of 65 with recent negative smears and why the low risk of invasive disease at the time of leaving the programme rapidly becomes attenuated with time from last negative smear^29^.

As to how some HPV integrants are silenced only to be re-expressed in later life is not known. Given that foreign DNA is frequently *de novo* methylated following insertion into the host genome, integration-associated changes in the viral epigenome may be a promising line of inquiry^30^. Histologically normal cells adjacent to HPV16 induced cervical lesions are reported to contain methylated and transcriptionally silent genomes which the investigators considered inactive or “silent” passengers^23^. Moreover, we have shown using longitudinal observations that viral integration is a common occurrence in young women following an incident cervical HPV infection and not as was once thought a late event in cervical carcinogenesis^13^,^31^. Re-expression of previously silent integrants following treatment with the demethylating agent, 5-azacytidine, has been shown in a cervical cancer cell line containing multiple integrated copies of HPV16^32^. Therefore, one possible unifying hypothesis is that the genome wide demethylation changes which occur as part of the ageing process lead to the transcriptional reactivation of methylated and silent HPV integrants^32^. Such a mechanism would be consistent with longitudinal studies showing that global DNA hypomethylation increases the risk of developing cancer at different sites^33^,^34^. Of course, the persistence in cervical epithelium of transcriptionally silent HPV integrants, or of disrupted but not integrated forms of the viral genome, which in themselves confer no obvious survival advantage would be dependent on their continuing presence in a stem cell compartment; this has yet to be demonstrated.

While our study provides evidence of disrupted forms of high-risk HPV DNA in morphologically normal cervices, it will be important to recapitulate these findings in a validation cohort using both FFPE and fresh tissue collected from women undergoing hysterectomy for reasons unrelated to epithelial abnormality. In addition to replicating our current findings, fresh tissue should be used to characterise the HPV DNA in terms of methylation status, and confirm physical status by identification of specific integration sites in the host genome using a next generation sequencing technology.

Our findings point to a reservoir of transcriptionally silent disrupted HPV16 genomes in morphologically normal cervices and re-activation of these “latent” forms of HPV DNA could explain the increase in incidence of cervical cancer observed in later life. A recent report has argued a case for extending cervical screening to women older than 65^35^; we believe our findings will contribute to this debate especially when falling hysterectomy rates and longer life expectancy in a population which is more heavily HPV exposed than previous generations, mean that more cervices will be at risk for longer.

## Additional Information

**How to cite this article**: Leonard, S. M. *et al.* Evidence of disrupted high-risk human papillomavirus DNA in morphologically normal cervices of older women. *Sci. Rep.* **6**, 20847; doi: 10.1038/srep20847 (2016).

## Supplementary Material

Supplementary Information

## Figures and Tables

**Table 1:** Validation of ISH results using PCR based methods.

	Luminex Positive N = 27	Luminex Negative N = 30	Chi Square (pearsons)	p Value	relative risk	95% Confidence Interval
Ventana Positive	8	0	10.34	0.0013		
Ventana Negative	19	30				
	Q-PCR Positive N = 24	Q-PCR Negative N = 24				
Ventana Positive	18	5	14.11	<0.0001	3.3	1.6–6.7
Ventana Negative	6	19				

For the Luminex assay, 76 samples were tested for HR-HPV types and 57 provided a usable result which could be compared to Ventana ISH results. When we compared the overlap between Ventana positive and Luminex positive samples we found 8/27 cases were HPV positive using both methods. When we compared the overlap between Ventana negative and Luminex negative samples we found 30/30 cases were HPV negative using both methods. When we compared the overlap between Ventana negative and Luminex positive samples we found 19/27 were negative using Ventana but positive using Luminex. When we compared the overlap between Ventana positive and Luminex negative samples we found 0/30 were positive using Ventana but negative using Luminex. Using a chi square test (pearsons) we found Ventana ISH positive samples were significantly more likely to test positive for HPV using Luminex (p = 0.0013). For the nested E6 Q-PCR assay, 55 samples were tested for HPV 16 and 48 provided a usable result which could be compared to Ventana ISH results. When we compared the overlap between Ventana positive and Q-PCR positive samples we found 18/24 cases were HPV positive using both methods. When we compared the overlap between Ventana negative and Q-PCR negative samples we found 19/24 cases were HPV negative using both methods. When we compared the overlap between Ventana positive and Q-PCR negative samples we found 5/24 were positive using Ventana but negative using Q-PCR. When we compared the overlap between Ventana negative and Q-PCR positive samples we found 6/27 were negative using Ventana but positive using Q-PCR. Using a chi square test (pearsons) we found Ventana ISH positive samples were also significantly more likely to test positive for HPV using Q-PCR (p = < 0.0001).

**Table 2:** Concordance of results from HPV testing using all three methods.

	Ventana Positive N = 18		Ventana Negative N = 17
ISH^+^ Luminex^+^ Q-PCR^+^	5	ISH^−^ Luminex^−^ Q-PCR^−^	13
ISH^+^ Luminex^+^ Q-PCR^−^	2	ISH^−^ Luminex^−^ Q-PCR^+^	4
ISH^+^ Q-PCR^+^ Luminex^−^	9	ISH^−^ Q-PCR^−^ Luminex^+^	0
ISH^+^ Q-PCR^−^ Luminex^−^	2	ISH^−^ Q-PCR^+^ Luminex^+^	0

A total of 55 samples were tested for HPV using all three methods, with 35 samples providing an evaluable result. 18 cases tested HPV positive using Ventana ISH; 5 of these were positive using all three detection methods; 2 were positive using ISH and Luminex but negative using Q-PCR, 9 were positive using ISH and Q-PCR but negative using Luminex; 2 were positive using ISH but negative using Luminex and Q-PCR. 17 cases tested HPV negative using Ventana ISH; 13 of these were negative using all three detection methods; 4 were negative using ISH and Luminex but positive using Q-PCR.
