# Supplementary material for: Evidence of disrupted high-risk human papillomavirus DNA in morphologically normal cervices of older women
Source: Sci Rep. 2016 Feb 15;6:20847. doi: 10.1038/srep20847 (PMC4753489; doi:10.1038/srep20847)
Supplement: Supplementary Information [file srep20847-s1.pdf]

Evidence of disrupted high-risk human papillomavirus DNA in morphologically normal cervices of older women

Running title: Disrupted HPV DNA in normal cervices

Authors: Sarah M. Leonard[1*], Merlin Pereira[1*], Sally Roberts[1], Kate Cuschieri[2], Gerard Nuovo[3], Ramanand Athavale[4], Lawrence Young[5], Raji Ganesan[6], Ciarán B. Woodman[1]

A

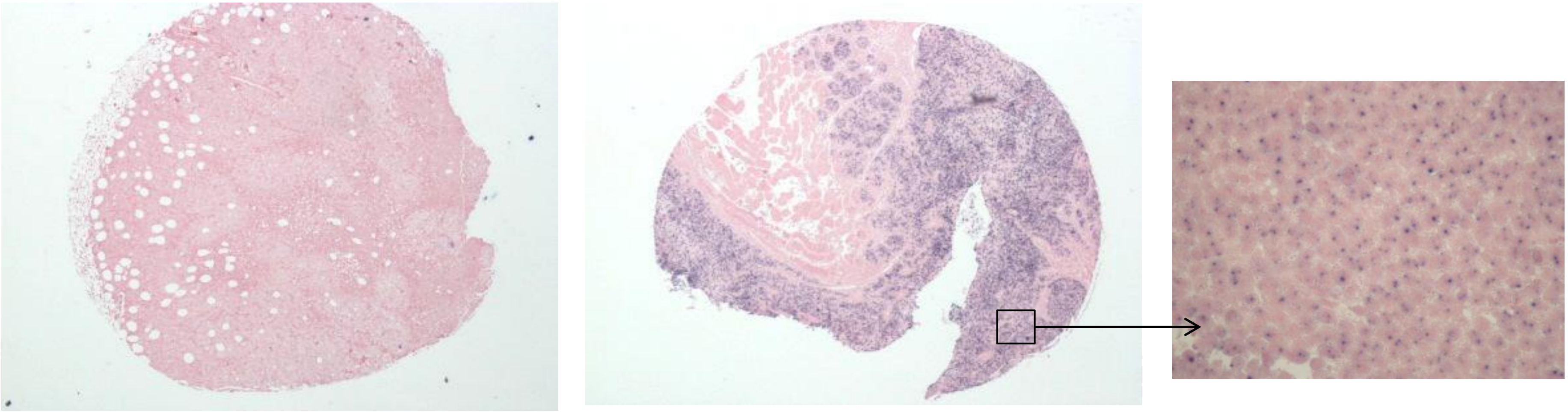

Negative control x10

Positive control x10

Positive control x40

B

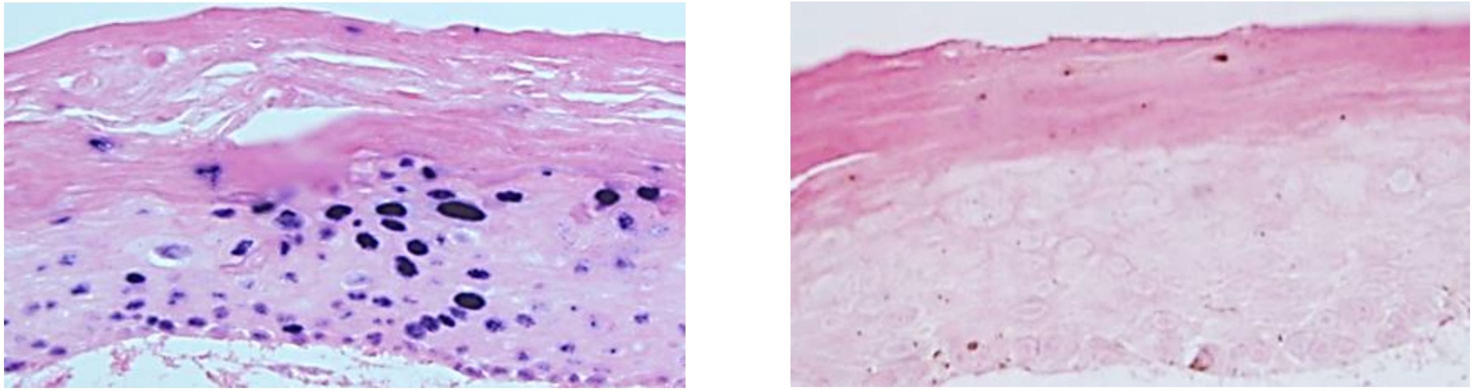

HPV 18 transfected keratinocyte raft tissue

Untransfected keratinocyte raft tissue

Supplemental Figure 1: Detection of high risk HPV in control tissue using Ventana ISH.

A: HPV untransfected (left) and transfected (right) xenograft mouse control tissue provided by Ventana. The left and middle panel show the results from Ventana ISH staining x 10 magnification and the right panel shows a higher magnification of the HPV positive tissue indicated by a box.

B: Positive and negative control tissue showing the presence of high risk HPV in HPV 18 transfected keratinocyte raft tissue.

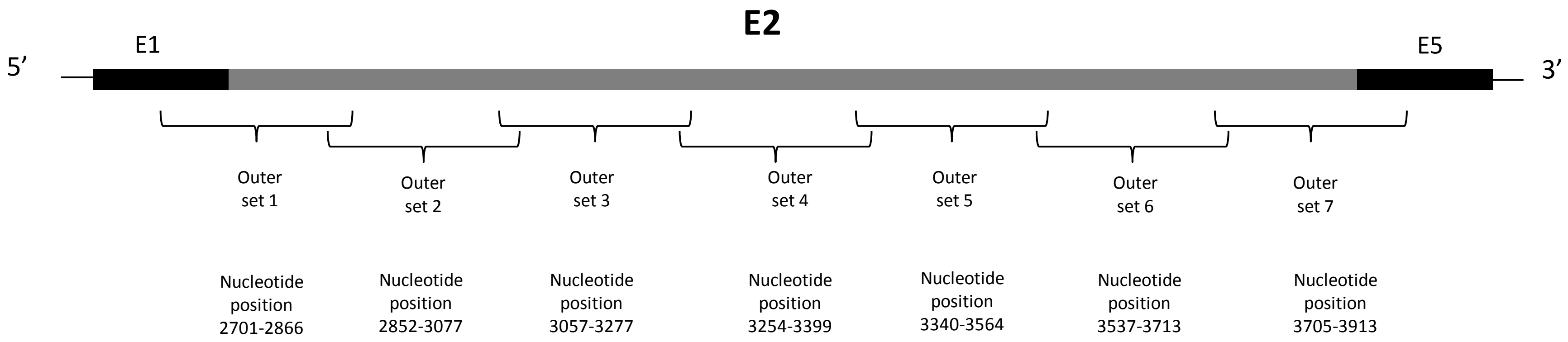

Supplemental Figure 2: Map showing the nucleotide location of where each of the overlapping primer sets are within the *E2* gene.

Supplemental Table 1 – List of primers used

Primers for nested E6 Q-PCR	PCR product size (bp)	Nucleotide positions
HPV16 E6 long set FWD 5’-GAACAGCAATACAACAAACC -3’		
HPV16 E6 long set REV 5’-GATCTGCAACAAGACATACA -3’	164	362-526
HPV16 E6 nested FWD 5’-CAAACCGTTGTGTGATTTGTT -3'		
HPV16 E6 nested REV 5’-TGAAGAAAAGCAAAGACATCTGG - 3'	80	381-461
HPV16 E6 probe 5' - TGTCAAAAGCCACTGTGTC - 3'		
Primers for Nested E2 PCR		
L - long outer primers		
S - short inner primers		
E2L1FWD 5’-AGGACGTGGTCCAGATTAAG -3’		
E2L1REV 5’ –TCTAGGCGCATGTGTTTCCA -3’	165	2701-2866
E2S1FWD 5’- TTAAGTTTGCACGAGGACGA -3’		
E2S1REV 5’-TTTAAACGTTGGCAAAGAGTCTCC -3’	60	2716-2716
E2L2FWD 5’ –CACATGCGCCTAGAATGTGC -3’	225	2852-3077
E2L2REV 5’ –ACATCCTGTTGGTGCAGTTA -3’		
E2S2FWD 5’ –TGCCAACACTGGCTGTATCA -3’	59	2925-2984
E2S2REV 5’ –AGTTGCAGTTCAATTGCTTGT – 3’		
E2L3FWD 5’ –TAACTGCACCAACAGGATGT – 3’	220	3057-3277
E2L3REV 5’ –TCTGCATCATCTTTAAACTGCACA – 3’		
E2S3FWD 5’ –CAATGCATTATACAAACTGGACACA – 3’	81	3132 -3213
E2S3REV 5’ CAACTTGACCCTCTACCACAGT – 3’		
E2L4FWD 5’ –GTGCAGTTTAAAGATGATGCAGA -3’		
E2L4REV 5’ –GCCAAGTGCTGCCTAATAAT – 3’	145	3254- 3399
E2S4FWD 5’ –ATCTGTGTTTAGCAGCAACG – 3’		
E2S4Rev 5’ –GCTGCCTAATAATTTCAGGAGAGG – 3’	52	3340-3392
E2L5FWD 5’-ATCTGTGTTTAGCAGCAACG -3’		
E2L5REV5’-TAAATGCAGTGAGGATTGGA -3’	224	3340-3564
E2S5FWD 5’ –CAGACGACTATCCAGCGACC – 3’		
E2S5Rev 5’ –AACTTAGTGGTGTGGCAGGG – 3’	65	3452-3517
E2L6FWD 5’-ACAGTGCTCCAATCCTCACT – 3’		
E2L6REV 5’- CCAATGCCATGTAGACGACAC – 3’	176	3537 -3713
E2S6FWD 5’ –TGCTCCAATCCTCACTGCAT – 3’		
E2S6REV 5’ –CCGTCCTTTGTGTGAGCTGT – 3’	45	3541-3586
E2L7FWD 5’- GGCATTGGACAGGACATAAT -3’		
E2L7REV 5’-CAAAAGCACACAAAGCAAAG -3’	208	3703-3913
E2S7FWD 5’ –GCATTGGACAGGACATAATGTAAA – 3’		
E2S7REV 5’ –TCACGTTGCCATTCACTATC – 3’	75	3707-3782

Supplemental Table 2 – List of Samples tested

Sample number	Group	HPV nested PCR	Luminex	P16 tested	E4 tested	E2 tested
43	negative	INVALID	INVALID	tested		
25	negative	not done	INVALID	tested		
78	negative	not done	INVALID	tested		
81	negative	not done	INVALID	tested		
72	negative	negative	INVALID	tested		
18	negative	negative	INVALID	tested		
31	negative	negative	INVALID	tested		
10	negative	positive	INVALID	tested		
91	negative	not done	not done	tested		
102	negative	not done	not done	tested		
105	negative	not done	not done	tested		
93	negative	not done	not done	tested		
107	negative	not done	not done	tested		
95	negative	not done	not done	tested		
106	negative	not done	not done	tested		
92	negative	not done	not done	tested		
99	negative	not done	not done	tested		
103	negative	not done	not done	tested		
41	negative	negative	not done	tested		
59	negative	negative	not done	tested		
97	negative	negative	not done	tested		
38	negative	INVALID	negative	tested		
42	negative	INVALID	negative	tested		
58	negative	INVALID	negative	tested		
13	negative	not done	negative	tested		
29	negative	not done	negative	tested		
3	negative	not done	negative	tested		
1	negative	not done	negative	tested		
14	negative	not done	negative	tested		
67	negative	not done	negative	tested		
50	negative	not done	negative	tested		
11	negative	not done	negative	tested		
30	negative	not done	negative	tested		

Continued from above

Sample number	Group	HPV nested PCR	Luminex	P16 tested	E4 tested	E2 tested
85	positive	positive	negative	tested	tested	tested
84	positive	positive	negative	tested		tested
75	positive	positive	negative	tested	tested	tested
66	positive	positive	negative	tested	tested	tested
61	positive	positive	negative	tested		tested
54	positive	positive	negative	tested	tested	tested
52	positive	positive	negative	tested		tested
28	positive	positive	negative	tested	tested	tested
8	positive	positive	negative	tested		tested
65	positive	negative	negative	tested		
51	positive	negative	negative	tested		
100	positive	positive	not done	tested		tested
80	positive	positive	INVALID	tested		tested
79	positive	positive	INVALID	tested		tested
73	positive	positive	INVALID	tested		tested
76	positive	negative	INVALID	tested		
2	positive	negative	INVALID	tested		
98	positive	not done	negative	tested		
89	positive	not done	negative	tested		
88	positive	not done	not done	tested		
87	positive	not done	INVALID	tested		
68	positive	not done	negative	tested		
44	positive	not done	negative	tested		
39	positive	not done	negative	tested		
35	positive	not done	not done	tested		
34	positive	not done	negative	tested		
21	positive	not done	negative	tested		
96	positive	INVALID	not done	tested		
70	positive	INVALID	negative	tested		
63	positive	INVALID	positive	tested		

Continued from above

Sample number	Group	HPV nested PCR	Luminex	P16 tested	E4 tested	E2 tested
17	negative	not done	negative	tested		
62	negative	negative	negative	tested		
40	negative	negative	negative	tested		
45	negative	negative	negative	tested		
19	negative	negative	negative	tested		
64	negative	negative	negative	tested		
60	negative	negative	negative	tested		
12	negative	negative	negative	tested		
27	negative	negative	negative	tested		
9	negative	negative	negative	tested		
48	negative	negative	negative	tested		
90	negative	negative	negative	tested		
7	negative	negative	negative	tested		
6	negative	negative	negative	tested		
47	negative	positive	negative	tested		
46	negative	positive	negative	tested		
32	negative	positive	negative	tested		
22	negative	positive	negative	tested		
83	positive	positive	positive	tested	tested	tested
82	positive	positive	positive	tested	tested	tested
77	positive	positive	positive	tested	tested	tested
33	positive	positive	positive	tested	tested	tested
15	positive	positive	positive	tested	tested	tested
74	positive	negative	positive	tested		
26	positive	negative	positive	tested		
